# High relative frequency of SCA1 in Poland reflecting a potential founder effect

**DOI:** 10.1007/s10072-016-2594-x

**Published:** 2016-05-19

**Authors:** Wioletta Krysa, Anna Sulek, Maria Rakowicz, Walentyna Szirkowiec, Jacek Zaremba

**Affiliations:** Department of Genetics, Institute of Psychiatry and Neurology, Sobieskiego 9 Street, 02-957 Warsaw, Poland; Department of Clinical Neurophysiology, Institute of Psychiatry and Neurology, Warsaw, Poland

**Keywords:** Rare neurodegenerative disorders, Spinocerebellar ataxia (SCA), Founder effect

## Abstract

Spinocerebellar ataxias (SCAs) have irregular distributions worldwide. SCA1 is the most frequent in Poland, and no cases of SCA3 of Polish origin has yet been identified. In view of such patterns of SCAs occurrence, the relative frequency, geographical distribution and a possible founder effect of SCA1 were investigated. DNA samples of 134 probands with SCA1 and 228 controls were analysed. The genotyping of four markers, D6S89, D6S109, D6S274, D6S288, around the ATXN1 gene (SCA1) and sequencing of the selected variant of D6S89 were performed. The relative frequency of SCA1 was 68 %. The studied SCA1 pedigrees were irregularly distributed, with the highest concentration in Central Poland. Haplotyping revealed the association of ATXN1 gene mutation with a 197-bp variant of D6S89 marker (63 % of probands) and with a 184-bp variant of DS6274 (50.7 % of probands). Out of 61 SCA1 probands from Mazowieckie, 41 carried the same 197-bp variant. SCA1 relative frequency in Poland shows the highest value compared with the data from other countries worldwide. Due to the association with the mutation obtained for the investigated markers and the SCA1 pedigrees concentration in Central Poland, we hypothesise that it represents a potential founder effect.

## Introduction

Spinocerebellar ataxias (SCAs) belong to a group of hereditary neurodegenerative disorders that represent a wide spectrum of clinical and neuropathological symptoms. Due to their approximated worldwide prevalence of 3:100,000, SCAs are assumed to be rare disorders. SCA3 is the most common subtype worldwide, followed by SCA1, 2, and 6. Depending on the geographical region, these 4 types account for approximately 75 % of all SCA familial cases. [1, 2].

The prevalence of particular subtypes differs in various ethnic groups and continents. The most spectacular examples are Holguin Province in Cuba, where SCA2 occurs at an estimated frequency of 41 cases per 100,000 individuals [3, 4] and Flores Island, which belongs to the Azores Archipelago, where up to 714 SCA3 cases per 100,000 were observed [5]. Such regional differences of the occurrence of some SCA types are the result of a founder effect, a phenomenon documented in a few populations, for example in Brazilian, Portuguese and Japanese patients for SCA3 [6, 7], in Scandinavian patients for SCA7 [8] and Mexican patients for SCA10 [9].

Contrary to the highest SCA3 frequency in the majority of Western European countries, in Poland and the Czech Republic, no cases of this SCA type have yet been identified among patients originating from these two countries [10, 11]. In the Czech Republic, the absence of SCA3 might result from lower frequencies (less than 0.1 %) of large normal alleles carrying >27 CAG repeats in the Czech control group (the range 13–34 CAG repeats) compared with Caucasians and other genetic factors, such as the presence or absence of certain haplotypes. Similarly, the genotyping of the *ATXN3* gene in Polish controls also revealed a lower normal range (14–32 CAG repeats) compared with other Caucasians (14–40 CAG repeats) [12, 13]. In contrast to the SCA3 *locus*, the range of 25–38 CAG and the mean allele size of 31.15 CAG triplets for the SCA1 (*ATXN1)* gene were slightly higher in the Polish group than in other genotyped Caucasian individuals (25–37 CAG, mean allele size 30.10).

In addition to 138 SCA1 pedigrees in Poland, 23 SCA2, 1 SCA3 (one pedigree originating from Germany), 38 SCA8 and 3 SCA17 pedigrees were identified.

Considering this pattern of SCA types occurrence in Poland, we attempted to determine whether these effects reflected the founder effect for SCA1.

## Materials and methods

The statistical analyses of the relative SCA1 frequency in Poland and investigation of its geographical distribution were performed for 138 unrelated probands representing 138 different families (previously verified SCA1 mutation carriers). The haplotyping analyses were carried out for 134 DNA samples of SCA1 probands. The control group comprised 228 healthy unrelated individuals (ages ranging from 19 to 96 years, mean age of 52 years). DNA samples were extracted from peripheral blood leukocytes using a standard phenol/chloroform method or automated isolation on a MagNA Pure Compact (Roche Instrument Center AG, Rotkreuz, Japan). The molecular methods for microsatellites repeats genotyping involved polymerase chain reaction (PCR) and electrophoresis in 5 % polyacrylamide gels of fluorescently labelled PCR products with the internal size marker TAMRA350 on an ABI Prism 377 plate sequencer (Applied Biosystems, Foster City, CA, USA) under denaturing conditions. The primer sequences used for genotyping and sequencing have been described previously: D6S89 [14], D6S109 [15], D6S274 [16], D6S288 [17].

Sequencing of the D6S89 CA polymorphism was performed using the BigDye Terminator v.3.1, followed by electrophoretic separation on the genetic analyser ABI3110 (Applied Biosystems/Hitachi, Tokyo, Japan). To determine the number of CA repeats in a 197-bp variant, sequencing was performed in the 5 DNA samples of SCA1 individuals.

Differences in the allele distribution between control and carrier chromosomes were evaluated using *X*^2^ analysis and Fisher’s exact test. The presence of LD was tested using HWE LR Chi-square for genetic markers (Statistical Genetic Utility Programs by J. Ott v.2007). A difference was considered statistically significant if *p* < 0.001.

The geographic distribution of the 138 SCA1 pedigrees was determined according to the data from files archived in the Genetics Department, Institute of Psychiatry and Neurology, Warsaw, Poland. The first criterion used to designate the region of origin of the SCA1 family was the place of birth of the oldest affected family member, and the second criterion, when the first was unavailable or unknown, was the address of the permanent residence. The region of descent was determined for 127 pedigrees: 120 pedigrees originated from Poland, 7 pedigrees originated from eastern neighbouring countries, and no data were available for the remaining 11 pedigrees. The administrative division of the country into 16 voivodships corresponded to the regions of origin.

Informed consent was obtained from all participants. This study was approved by the Bioethical Commission of The Institute of Psychiatry and Neurology, Warsaw.

## Results

### Relative SCA1 frequency

Relative frequency of SCA1 (68 %) was assessed considering the number of SCA1 pedigrees (138 probands) versus the overall number of molecularly confirmed ADCA pedigrees: 138 SCA1, 23 SCA2 1 SCA3, 38 SCA8 and 3 SCA17 (203 probands/families). A similar frequency (67 %) was obtained considering all SCA1 mutation carriers (258) versus all molecularly verified subjects with the types of ADCA described above (383).

### Geographical distribution of SCA1 pedigrees in Poland

**Fig. 1:**
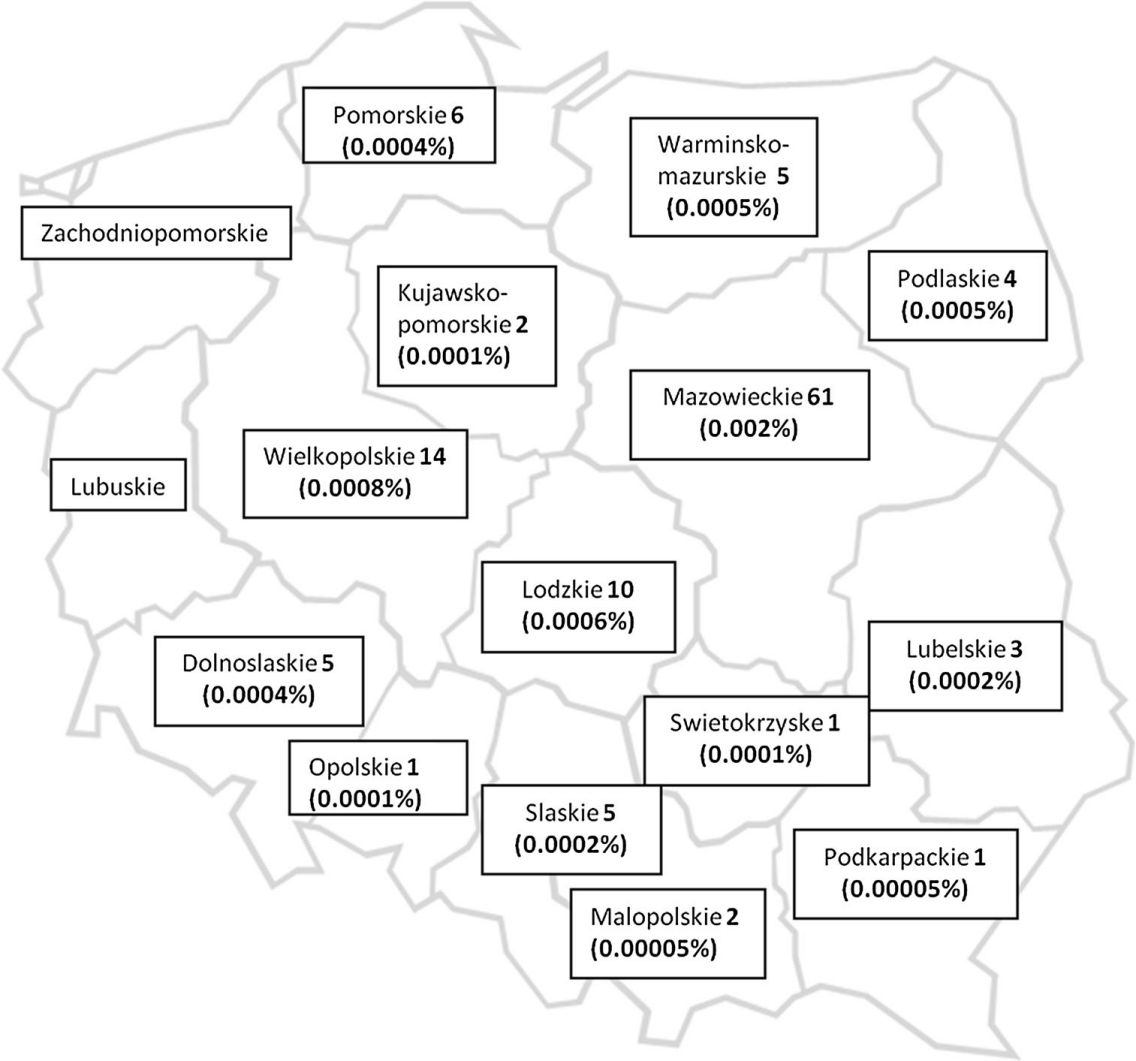
Geographic distribution of 120 SCA1 pedigrees of documented Polish origin. The number of SCA1 pedigrees (probands) is shown in the frame with the name of the voivodship, and the prevalence of SCA1 mutation carriers (symptomatic and asymptomatic) calculated per voivodship population is shown in *parentheses*

According to the geographical distribution study, 61 pedigrees affected with SCA1, accounting for 48 % of the analysed pedigrees, originated from Mazowieckie voivodship, where 13.7 % of the total Polish population resided, in concordance to the Statistical Yearbook of the Regions [18]. The other two regions of Poland, centrally located, where SCA1 cases were relatively numerous were Wielkopolskie (14 pedigrees) and Lodzkie (10 pedigrees). Among 138 pedigrees, 88 pedigrees (63.8 %) originated from the above named 3 voivodships in which SCA1 was more frequently observed. Seven pedigrees were of foreign origin: four pedigrees from Ukraine, two pedigrees from Russia and one pedigree from Belarus. Figure 1 provides the distribution of the 120 SCA1 pedigrees of Polish origin, and the percentage of all SCA mutation carriers identified (symptomatic and asymptomatic) in the voivodships is indicated in parentheses. Notably, although the largest population is located in Mazowieckie (5293.2 thousands), the highest percentage of SCA1 mutation carriers (0.002–129 SCA1 mutations) was observed, which suggests that SCA1 prevalence may be about 1: 41.031 (~2.5:100,00). In the other voivodships, the prevalence of the mutated *ATXN1* gene carriers is at least 10 or 100 times lower compared with Mazowieckie. The assessed overall mean prevalence of SCA1 in Poland is approximately 1:100,000 individuals (assuming that most of the affected pedigrees have been identified).

### Haplotyping study

The dinucleotide polymorphisms of genetic markers spanning the region around *locus ATXN1* (SCA1), D6S89, D6S109, D6S274, and D6S288, were analysed in the group of individuals carrying the CAG repeat expansion and controls. The obtained results established the allele frequencies for each marker to determine whether any disequilibrium existed between the studied groups. The analysed variants frequencies (calculated for alleles) are shown in Table 1.

**Table 1:** Allele frequencies of the analysed markers in the studied group of 134 SCA mutation carriers and 228 controls and statistical significance of the differences between the groups studied

Locus	Allele (bp)	Expanded chromosomes^a^ (frequency)	Non-expanded chromosomes (controls^b^) (frequency)	*Χ* ^2^	*p*
D6S89	197	86/268 (0.321)	37/456 (0.081)	68.80	0.00001
	193	54/268 (0.201)	108/456 (0.237)	1.21	0.2705
D6S274	184	80/268 (0.298)	36/456 (0.079)	60.48	0.00001
	174	44/268 (0.164)	130/456 (0.285)	13.52	0.0002
D6S109	185	99/268 (0.369)	192/456 (0.421)	1.87	0.1711
D6S288	234	129/268 (0.481)	215/456 (0.471)	0.07	0.7978
	232	79/268 (0.295)	105/456 (0.230)	3.71	0.05663

^a^The studied group comprised 134 SCA1 mutation carriers (probands) (268 alleles)

^b^The control group comprised 228 unaffected individuals (456 alleles)

Further statistical analyses (Table 2) were performed for the allelic variants of the two markers, which were selected according to a value of *p* = 0.001 and confirmed the significant differences between both studied groups (calculated for the subjects studied). Association was calculated using Fisher’s exact test, and a *p* value <0.001 was considered significant. The first allele, corresponding to the 197-bp variant of D6S89, was overrepresented (*p* < 0.001) in the carriers of the expanded chromosomes compared with unexpanded chromosomes. Moreover, of the 61 probands from Mazowieckie voivodship, the 197-bp allele was detected in 41 probands. For the second D6S274 marker an allelic variant, corresponding to the 184 bp, was also more frequent in SCA1 individuals than in controls (Table 2). Overall, the haplotype D6S89/D6S274 (197/184 bp) was observed in 46 probands among 134 individuals studied (34 %) versus the control group, where of the 228 individuals analysed, 8 individuals (3.5 %) carried the same variants. Additionally, among the 46 subjects with SCA1 carrying the 184-bp variant of D6S274, 10 subjects were homozygous for this variant.

**Table 2:** Analysis of the association between the polymorphic marker variants and SCA1 in the group of mutation carriers analysed using Fisher’s exact test

Marker	Allele (bp)	Controls^b^	Mutation carriers^a^	LR likelihood ratio (2*p*)	Fisher’s test (*p*)	D’ linkage disequilibrium parameter
D6S89	197	37/228	84/134	0.0000	0.0000	−0.514
D6S274	184	36/228	68/134	0.0000	0.0000	−0.450
	174	130/228	37/134	0.0000	0.0000	+0.401

^a^The studied group comprised 134 SCA1 mutation carriers (probands)

^b^The control group comprised 228 unaffected individuals

Sequencing of the 197-bp allele sized through genotyping (fragment analysis) revealed the presence of 22 CA repeats.

## Discussion

According to the available epidemiological data, SCA1 is the most prevalent genetic type of SCA in Poland, as the relative frequency of SCA1 (68 %) and the number of identified pedigrees (138) is the highest compared with the data from other countries. Interestingly, the SCAs profile was most similar to that identified in Italy, with SCA1 as the most common type [19, 20]. Based on the Italian data, the SCA1 relative frequency in this country ranges from 13.9 % [21], 31.8 % [22], 42.9 % [20] to 58.9 % [19] and also shows the irregular distribution, with significantly higher density of SCA1 pedigrees in the northern part of the country. The other country where SCA1 seems to be more prevalent is Russia, with a relative frequency of 33 % [23], although it might be underestimated, due to the small number of families studied.

Similarly to Italian studies, the SCA1 distribution in Poland is irregular, with the highest concentration of pedigrees occurring in Mazowieckie voivodship [11].

We assumed that these results partially reflect the selection bias which was caused by availability of genetic testing for SCAs restricted at the time of the study to the Genetic Department (IPN Warsaw). Additionally, such irregular distribution of SCA1 may have also resulted from limited access to specialised medical centres throughout the country. However, the analysis of the geographical distribution of SCA1 families, especially those with at least three generations, revealed their actual concentration in the Central Poland (Mazowieckie). Moreover, their members were not in favour of changing residence. When changes in residency were observed, moving out the place of family origin was primarily confined to the same region. Although Mazowieckie has the largest population (5293.2 thousands of inhabitants), the highest prevalence (0.002 %) of SCA1 mutation carriers was found.

Additionally, the haplotyping study revealed that among the 61 SCA1 probands from this region, 41 probands carried the same 197-bp variant of the D6S89 marker, which is strongly associated with the *ATXN1* locus. This finding suggests that the mutation might have derived from this region, thus representing an example of the founder effect.

The haplotyping within the group of Polish SCA1-affected families revealed two statistically significant associations: the 197-bp D6S89 marker variant, coinherited with the mutation in 84 pedigrees, and the 184-bp D6S274 marker variant, coinherited with the mutation in 64 pedigrees. However, both variants were identified only in 46 probands. In view of the relatively high number of individuals homozygous for the 184-bp DS6274 variant, we assumed that this result does not show a considerably strong association with the SCA1 causative mutation. Additionally, the region size between these polymorphisms was approximately 1.2 Mb [16]; probably therefore, a relatively low number of probands carrying the haplotype was identified in the studied group.

**Fig. 2:**
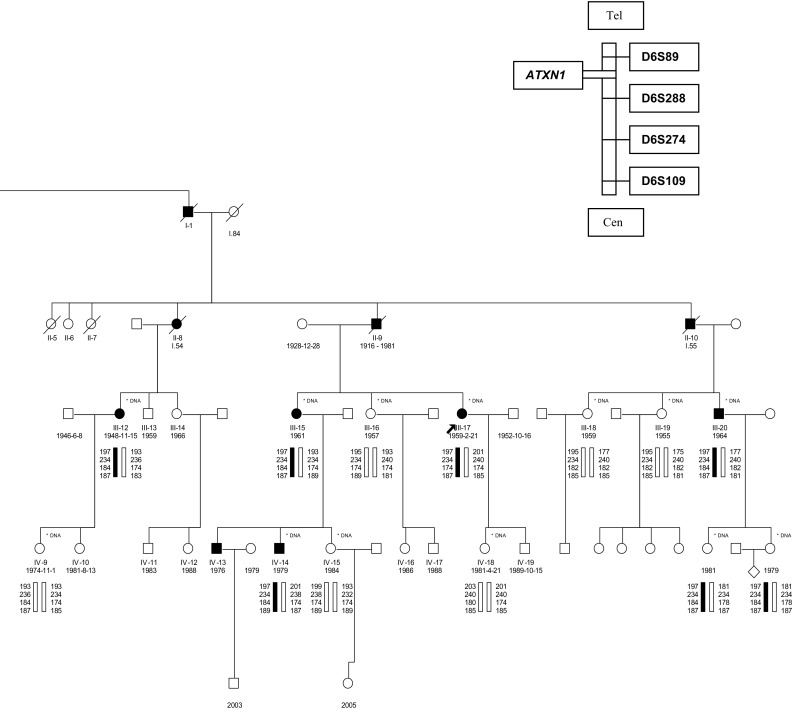
An example of the SCA1 pedigree with the 197-bp D6S89 variant segregating with the mutation

Comparing to our study, different methodological approaches in genotyping were found in majority of the reviewed articles. In previous studies, the size of D6S89 allelic variants was based on PCR amplification with radiolabelled primers and visualisation of amplicons on films after exposure to 4 or 6 % polyacrylamide denaturing gels [24–26]. Moreover, several studies have only reported the number of associated markers, without revealing the size in base pairs and data concerning the number of CA dinucleotides within these polymorphisms [27, 28]. Therefore, in the present study sequencing analysis of the 197-bp variant of the D6S89 marker was performed in five members of the two largest families, in whom this variant was coinherited with the SCA1 mutation, as shown in Fig. 2. We have determined that the 197-bp allele sized through genotyping contained 22 CA repeats.

Considering these different methodological approaches, we had limited information to compare the investigated polymorphisms according to the size in base pairs and/or to the sequence.

The search for available reports revealed that the same D6S89 marker variant of 197 bp was associated with the SCA1 mutation in 2 large Italian families originating from Calabria [24].

In all affected subjects (83 individuals) in both families, similarly to our study, the same D6S89 (197 bp) variant was identified. The authors suggested that this variant originated from a common ancestor according to family studies revealing the potential relationship dating back to the early 1700s. This assumption is supported by the low recombination frequency (less than 1 %) between both loci: SCA1 and D6S89 [29]; therefore, this dinucleotide polymorphism has been considered a highly informative marker prior to the SCA1 gene identification and was recommended for use in linkage analyses in the affected families [30, 31]. For example, in the study of Khati et al. [25], involving ten families with ADCA type 1, the linkage with the D6S89 was observed in four families. However, the analysed marker variants (209, 205, 201, and 193 bp) coinherited with the mutation were different in every pedigree. The authors postulated that this result might reflect the independent occurrence of the SCA1 mutation in these four kindreds originating from different regions of France.

Jodice and co-workers performed haplotyping to delineate the SCA1 *locus* in eight Italian kindreds [26]. The established haplotype segregating with the disease locus included four microsatellite markers, D6S288 (233 bp), D6S89 (197 bp), D6S260 (157 bp), and D6S289 (223 bp), considered as specific to the southern region of the country.

Another genotyping study of two SCA1 flanking polymorphisms, D6S89 and D6S274 [32], in seven pedigrees of the Siberian Yakut population revealed an association with the disease locus. Moreover, Goldfarb et al. previously described the founder effect in Yakuts [33], but no molecular analyses were performed at that time. Despite the relatively low number of subjects (7 families with 17 mutation carriers), informative data were obtained for both microsatellites, showing an association of a 174-bp allele (D6S274) in five pedigrees, a 199-bp allele (D6S89) in five families and a 205-bp allele in one family. The authors postulated that the two independent historical recombination events between the SCA1 gene and D6S89 marker resulted in a switch to the allele of 205 bp which may be consistent with the different regions of origin documented for two families from Southern and Northern Siberian villages.

## Conclusion

In Poland, the relative frequency of SCA1 among other molecularly identified types of SCA was 68 %, showing the highest value compared with the data from other countries in Europe and worldwide.

The distribution of SCA1 in Poland is irregular. The highest concentration of SCA1 pedigrees was observed in the centrally situated Mazowieckie voivodship. Haplotyping analyses revealed the association of *ATXN1* gene mutation with a 197 bp (bearing 22 CA repeats) D6S89 marker variant (in 84 out of 134 probands—63 %). Among the 61 SCA1 probands from Mazowieckie, 41 probands carried the same 197-bp variant. We hypothesise that the mutation might have occurred in the Central Poland region and that it represents a potential founder effect.
